# Jugular Vascular Closure and Scar Formation after Leadless Pacemaker Implantation

**DOI:** 10.31083/j.rcm2512440

**Published:** 2024-12-16

**Authors:** Shmaila Saleem-Talib, Crispijn P. R. Hoevenaars, Vincent J. van Driel, Harry van Wessel, Jeroen van der Heijden, Hemanth Ramanna, Natasja M. S. de Groot

**Affiliations:** ^1^Department of Cardiology, Haga Teaching Hospital, 2545AA The Hague, The Netherlands; ^2^University of Applied Sciences of The Hague, 2521EN The Hague, The Netherlands; ^3^Department of Cardiology, Erasmus Medical Center, 3015GD Rotterdam, The Netherlands

**Keywords:** jugular vein, Micra, leadless pacemaker, Perclose ProGlide, vascular closure device, large bore vascular access

## Abstract

**Background:**

Achieving hemostasis of large bore venous access sites can be challenging and time consuming. Closure devices have proven to be superior in achieving hemostasis, reducing time to ambulation and improving patient comfort, compared to manual hemostasis techniques after femoral venous and arterial access. The closure of the jugular vein following large bore access has not been investigated in previous studies. In addition, scar formation of the neck after large bore access of the jugular vein has not been investigated. In this study, the safety and feasibility of the double Perclose ProGlide (PP), for achieving hemostasis of the internal jugular vein (IJV) following large bore access with 27 French Micra Transcatheter Pacemaker System (TPS) was examined. Also, the scar formation in the neck after IJV closure was examined during follow-up.

**Methods:**

136 consecutive patients from May 2018 until June 2024, in whom the IJV was closed with a double PP, following Micra TPS implantation were included. All patients were examined for hemostasis of the IJV and vascular complications, resulting in additional interventions. Time to ambulation, discharge and patient discomfort were also assessed. During follow-up the scar formation of the neck was examined.

**Results:**

In all patients, the double PP was successful in achieving acute hemostasis of the IJV after large bore access. In all patients, 2 PP were deployed without device failure. One patient required additional manual pressure due to a minor hematoma. Ultrasound guided examination did not reveal any vascular complications. All patients were ambulated immediately. During follow-up, the scar in the neck was hardly visible.

**Conclusions:**

Although the PP was designed as a closure device for femoral venous and arterial access, our data suggest that the PP can be used safely as a closure device for the IJV to achieve acute hemostasis, facilitate direct ambulation and improve patient comfort.

## 1. Introduction

The number of interventions requiring large bore vascular access have rapidly increased in the last decade, due to interventions such as leadless pacemaker (LP) implantation [1, 2], transcatheter aortic valve replacement (TAVR) [3, 4, 5], transcatheter mitral valve repair (Mitraclip) [6] and mechanical circulatory support with extracorporeal membrane oxygenation (ECMO) or Impella [7, 8].

All these emerging interventions require large bore venous or arterial access, emphasizing the importance of obtaining hemostasis and limiting vascular complications. In addition, it is desirable to promote early patient ambulation and discharge, reduce the duration of anticoagulant therapy and increase patient comfort [9]. The Perclose ProGlide (PP) (Abbott Vascular, Santa Clara, CA, USA) is a suture-mediated vascular closure device, developed for femoral vein and femoral artery puncture site closure, after small and large bore interventions with a maximum 29 French (Fr.) outer diameter for venous sheaths. The PP was initially designed as a closure device for the femoral artery. More recently, the Food and Drug Administration (FDA) also approved this device for femoral vein closure [10]. At present, studies on closing the internal jugular vein (IJV) after large bore access are lacking and there is no information on the visibility of scar formation in the neck after large bore access. We therefore aimed to study the safety and feasibility of the Double PP with a preclose technique for achieving hemostasis of the IJV after LP implantation and to examine the visibility of scar formation in the neck during follow up.

## 2. Methods

### 2.1 Study Design

This is a single center prospective observational study of all consecutive patients, in whom the right IJV was closed with double PP after large bore access 27 Fr. following Micra Transcatheter Pacemaker System (TPS) implantation. Data collection was performed retrospectively and the usage of a vascular closure device after large bore vascular access is standard practice at our institution. Participation in this study did not influence the decision to use a vascular closure device. In 2018, the PP was introduced as a vascular closing device in our center. All operators were certified by the manufacturer to use the device for the femoral artery and vein. Starting from May 2018 till June 2024, 138 consecutive patients were included. In the first two patients the IJV was closed using a figure of 8 suture due to unavailability of the PP, therefore these two patients were excluded from our analysis. All other consecutive patients, consisting of 136 patients, were included in this study. The jugular approach is the preferred approach in our center due to its shorter route to the right atrium, resulting in easier manipulation of the catheter, making a higher right ventricle (RV) septum implantation easier. This results in a narrower paced QRS complex and reduces the risk of pacing-induced cardiomyopathy; all the benefits of the jugular approach have been described in our previously published papers [11, 12, 13]. Adequate acute hemostasis of the IJV was observed by the implanting cardiologist following implantation. Vascular complications were defined as hematoma, bleeding requiring manual pressure blood transfusion or any other puncture site related complications, time to ambulation, time to discharge and patient discomfort were documented before discharge. This study was approved by the regional medical ethical committee, the hospital’s scientific committee and the hospital’s medical board of directors. All patients gave informed consent to the procedure.

### 2.2 Double Perclose ProGlide with Preclose Technique

Direct oral anticoagulant (DOAC) therapy was discontinued for 24 hours before LP implantations. In patients using a vitamin K antagonist (VKA) the international normalized ratio (INR) level was adjusted between 2.0–2.5. No patients required bridging of anticoagulant therapy. Any antiplatelet therapy was continued. After the 27 Fr. sheath was inserted, a 2500 international units (IU) Heparin bolus was administered.

**Fig. 1.**
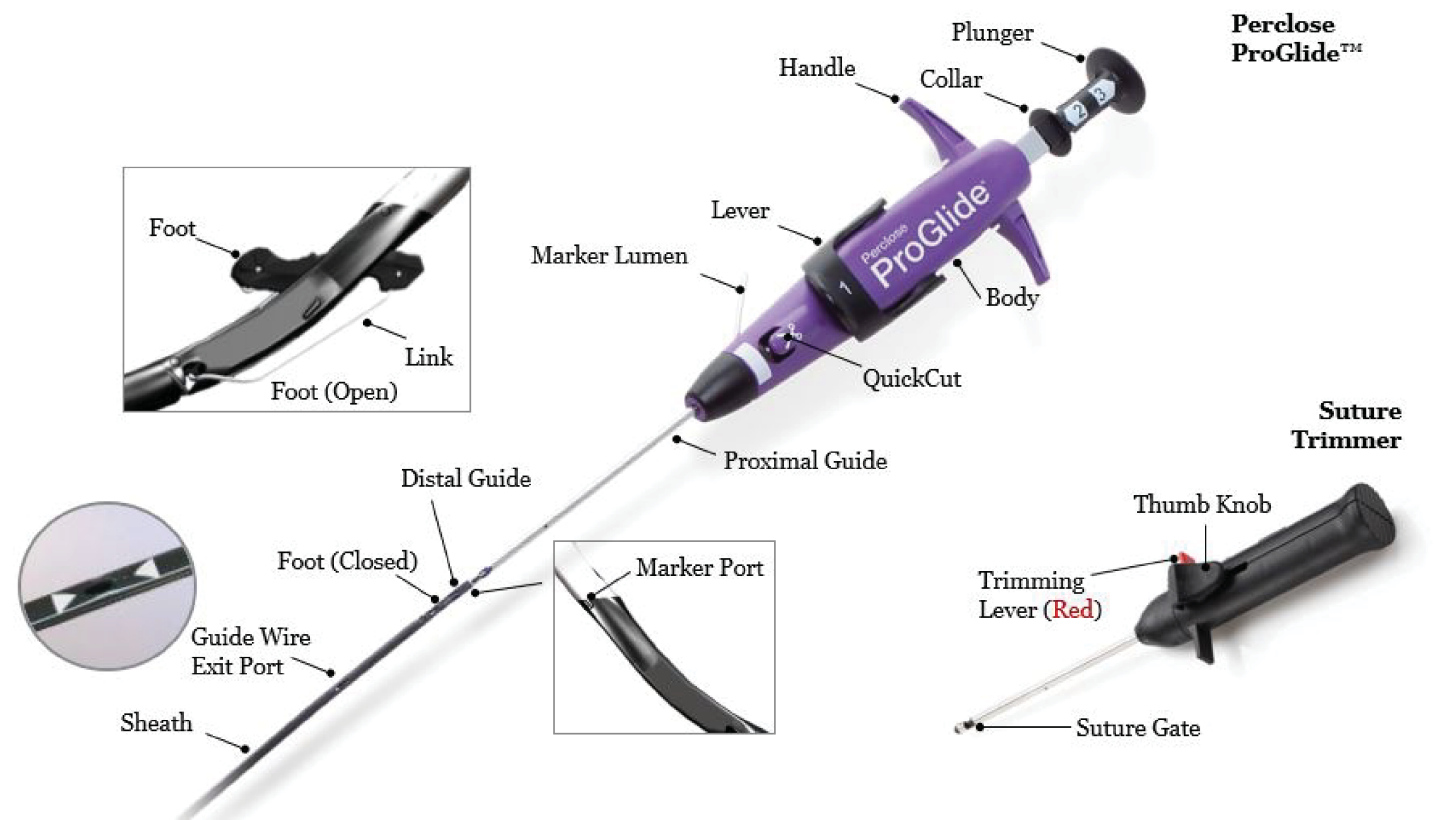
**Perclose Proglide a suture-mediated vascular closure device, developed for femoral vein and femoral artery puncture site closure after large bore access up to 29 French**.

**Fig. 2.**
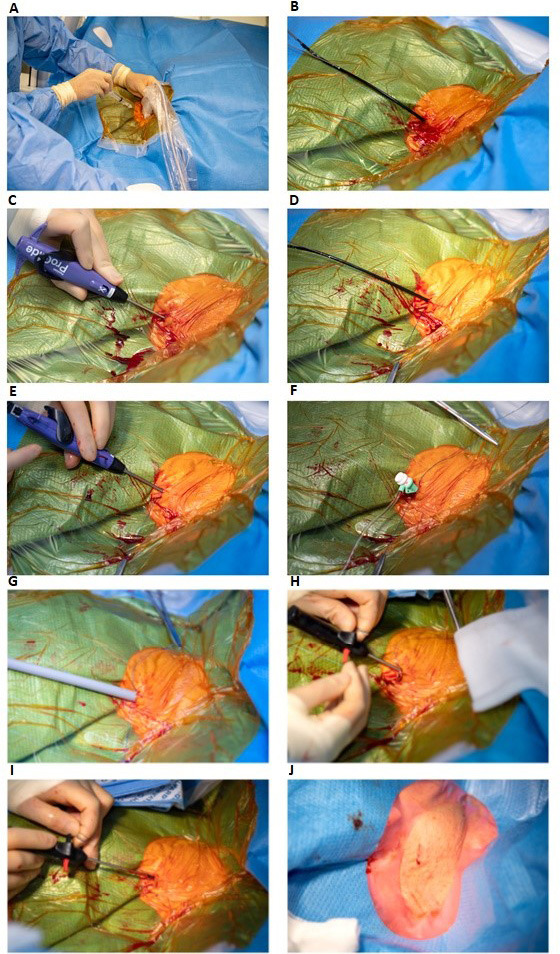
**Double Perclose ProGlide technique for closing the internal jugular vein after large bore access with a 27 Fr. Micra TPS delivery tool**. (A) Ultrasound guided puncture of right internal jugular vein. (B) Placement of the first Perclose ProGlide over the guide wire in a 45° degree angle in the right internal jugular vein. (C) Rotation of the first Perclose ProGlide to a 10:00 o’clock position and the first Perclose ProGlide is deployed. (D) The first suture is secured with a hemostat. (E) The second Perclose ProGlide is inserted and rotated to a 02:00 o’clock position, the second Perclose ProGlide is deployed and the second suture is secured with a hemostat. The double Perclose Proglide are paced perpendicular to each other. (F) A six Fr. sheath is reinserted over the guide wire. (G) The 27 French Micra TPS delivery tool is inserted in the internal jugular vein after serial dilation with a 16 French, followed by an 18 French dilator over a stiff wire. (H) After the Micra has been implanted in the right ventricle, the Micra TPS delivery tool is removed with simultaneously pulling the first placed Perclose ProGlide rail and advancing the suture trimmer to lock the knot. (I) This was repeated for the secondly placed Perclose ProGlide with the suture trimmer locking the second knot. (J) Acute hemostasis is observed. Fr., French; TPS, Transcatheter Pacemaker System.

In the first 15 patients, the IJV was cannulated using the technique described by Daily, Griepp and Shumway using the 2 heads of the sternocleidomastoid muscle as anatomical landmarks [14]. In the following 121 patients, the IJV puncture was guided by ultrasound, this was due to the availability of a linear echo probe. The venous position of the wire was confirmed with ultrasound and fluoroscopic imaging showing the wire passing through the right atrium into the inferior vena cava. After confirmation of a successful venous puncture without inadvertent arterial entry, the skin and subcutaneous tissue were incised transversally in the preexisting skin line. After dilation of the puncture site with a 6 Fr. sheath, the first PP was inserted into the IJV over the guidewire at a 45° angle (Figs. 1,2). When the guidewire exit port reached skin level the guidewire was removed. The PP was advanced until venous blood return was observed from the marker lumen. Then the PP was rotated 30° towards the 10:00 o’clock position and the first PP was deployed. The suture was secured with a hemostat.

In the following step, the second PP was inserted over the guidewire using the same technique, except that the second PP was rotated 30° towards the 02:00 o’clock position and deployed. Hereby placing 2 sutures perpendicular to each other. Next, a 6 Fr. sheath was reinserted, and a long stiff wire was inserted into the inferior vena cava under fluoroscopic guidance. After removing the 6 Fr. sheath, serial dilation of the entrance was performed over the stiff wire with a 16 Fr. followed by an 18 Fr. dilator. Afterwards the 27 Fr. diameter Micra TPS delivery tool was advanced; the LP implantation method has been described in our previous study and in the guidelines for LP implantation [11, 12, 15]

After LP implantation, the delivery sheath was removed with simultaneously pulling the first placed PP rail and slowly advancing the suture knot. Then the suture trimmer was advanced to lock the knot. This was repeated for the second placed PP. Adequate hemostasis was observed directly (Figs. 1,2). Finally, a single non-absorbable superficial skin suture was applied, which was removed at the first outpatient clinic visit 1 week after the implantation. After the implantation procedure, all patients were monitored in the cardiac ward and could be ambulated immediately.

They were discharged from the hospital the same day if the LP measurements were satisfactory, the chest X-ray showed satisfactory position of the LP and no complications were observed. Anticoagulant therapy was reinstated 6–8 hours after LP implantation.

### 2.3 Follow-up

**Fig. 3.**
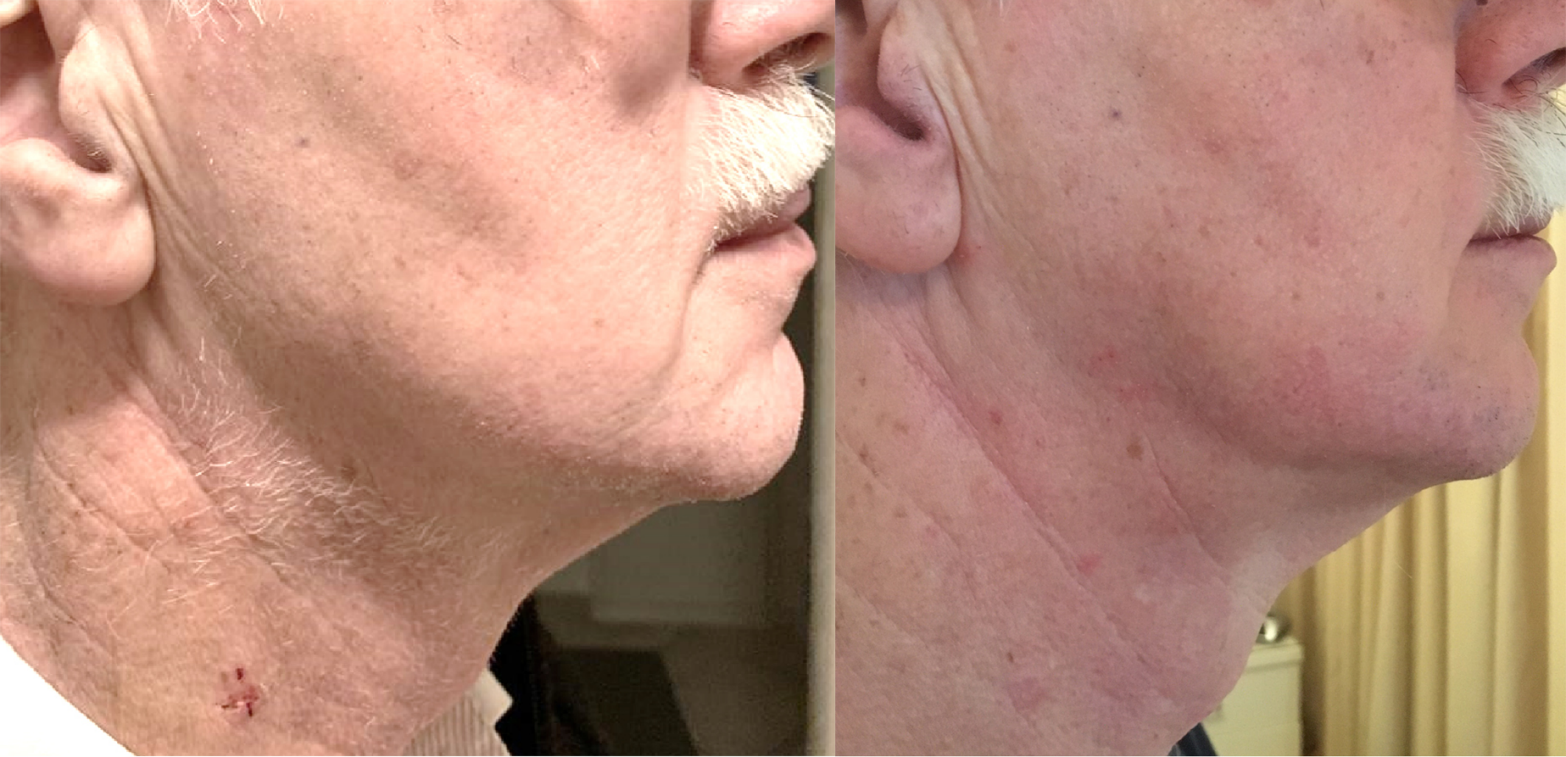
**Visibility of scar after jugular vein closure with Perclose Proglide**. On the left a photo of the scar one week after jugular vein closure with Perclose ProGlide, during the first outpatient clinic visit, where the skin suture is removed. On the right a photo of the same patient one month later during the outpatient clinic visit.

Vascular access site complications as described above, were reported directly after implantation in the electrophysiology (EP) lab, before discharge from the cardiac ward and during pacemaker follow-up visits at the outpatient clinic 1 week and 1 month after implantation. During these visits the scar formation in the neck was also examined (Fig. 3).

### 2.4 Statistical Analysis

Continuous variables were tested for normality using both the Kolmogorov-Smirnov and Shapiro-Wilk test. Normal variables were expressed as mean ± standard deviation (SD). Non-normally distributed variables were presented with median and range. Categorical variables are shown as percentages. Statistical analysis was performed using IBM SPSS statistics for Windows, version 28 (IBM Corp, Armonk, NY, USA).

## 3. Results

### 3.1 Patient Characteristics

Baseline characteristics of the patients (N = 136, 42% female, mean age at implantation: 79 years) are shown in Table 1.

**Table 1.** **Patient characteristics**.

Patients (N = 136)		
Age yrs, mean (range)		79 (17–100)
Follow-up mo, mean (range)		22 (0–67)
Female, n (%)		57 (42)
BMI, mean ± SD		27 (6)
Pacing indication, n (%)		
	Bradycardia	44 (32)
	AV-block	37 (27)
	AV-block post TAVR	2 (1)
	Pre-AVNA	39 (29)
	Micra replacement	2 (1)
	VVI PM dysfunction	5 (4)
	Pocket infection	7 (5)
Anticoagulant use, n (%)		
	None	10 (7)
	Antiplatelet	10 (7)
	DOAC	77 (57)
	DOAC + antiplatelet	13 (10)
	VKA	22 (16)
	VKA + antiplatelet	4 (3)
Comorbidities, n (%)		
	LVEF >55%, n (%)	98 (72)
	Atrial fibrillation	114 (84)
	Hypertension	114 (84)
	Hypercholesterolemia	67 (49)
	Diabetic	32 (24)
	Peripheral vascular disease	49 (36)
	Coronary artery disease	48 (35)
	Valvular heart disease	65 (48)
	Congestive heart failure	56 (41)
	COPD	25 (18)
	Pulmonary hypertension	34 (25)
	Renal dysfunction	22 (16)
	Congenital heart disease	6 (4)

Table 1: Values are reported as mean ± standard deviation (SD), n (%) or mean with range. AV-block, atrioventricular-block; BMI, body mass index; DOAC, direct oral anticoagulant; VKA, vitamin K antagonists; COPD, chronic obstructive pulmonary disease; LVEF, left ventricular ejection fraction; mo, months; Pre-AVNA, prior to atrioventricular node ablation; TAVR, transcatheter aortic valve replacement; VVI PM, pacemaker with VVI mode both sensing and pacing in the ventricle; yrs, years.

Due to the older age of our patient population, the majority of patients had multiple comorbidities, such as atrial fibrillation (84%), hypertension (84%), diabetes (24%), coronary artery disease (35%), peripheral vascular disease (36%) and valvular heart disease (48%).

Patients (N = 126 (93%)) had anticoagulant and/or antiplatelet therapy, which consisted of DOAC (57%) VKA (16%), antiplatelet therapy (7%) and dual therapy consisting of either DOAC and antiplatelet or VKA and antiplatelet therapy (13%). A detailed description of the indications for pacing, LP implantation and implantation outcomes have been described in our previous articles [11, 12].

### 3.2 Internal Jugular Vein Closure and Complications

In every patient, a double PP with a preclose technique was used to close the IJV, following large bore access with 27 Fr. Micra TPS. All PP deployments were successful, no device failures occurred and acute adequate hemostasis was observed. In one patient, a minor hematoma required light manual pressure for 10 minutes. Vascular complications were excluded by ultrasound imaging and there was no decrease in hemoglobin level. All patients were ambulated immediately. There were no access site bleedings. Patients did not require additional analgesics other than acetaminophen.

Although all patients were allowed discharge on the same day of the procedure, the majority of patients preferred an overnight hospital stay due to advanced age, only 36 (26%) patients were discharged the same day. Periprocedural, two complications occurred which are also described in our previous manuscript [11, 12]. One device dislodgement in which the device had to be snared and a new device was implanted in the right ventricular mid septum and one pericardial effusion which could be resolved with pericardiocentesis.

### 3.3 Follow-up at the Outpatient Clinic

All patients completed the one week follow up at the outpatient clinic, no vascular complications were observed and patients did not describe any discomfort in the neck. 18 patients were referred to us from other hospitals. However, the first follow-up visit at one week was always completed at our center. During which the puncture site could be examined and the non-absorbable superficial skin suture, which was placed at implantation could be removed. The one month follow up period was completed at their own hospital. 118 patients completed the one month follow-up at our center.

During these visits, the neck was examined for scar formation, in 117 patients the scar was not visible after one month. In one patient a scar was visible, because the skin incision was not performed in the skin lines. Therefore, a small scar can be seen outside of the skin line. During follow-up there was one patient with a threshold rise, which was resolved by increasing the output. Thereafter, the threshold remained stable.

## 4. Discussion

### 4.1 Major Findings

To our knowledge, this is the first study to investigate the closure of the IJV with the PP suture mediated vascular closing device, following large bore access for Micra TPS implantation with a 27 Fr. delivery sheath in a large cohort of 136 patients.

In all patients, 2 PP could adequately be deployed, without any device failures. In all patients, acute hemostasis was observed in the EP lab. There were no vascular complications, only one patient with a minor hematoma requiring additional manual compression. In almost all patients, there was no visible scar formation in the neck.

### 4.2 Clinical Evidence

So far, studies have described closure of the femoral vein with the PP after large bore access. Deshmukh *et al*. [1] describe the double PP for closing the femoral vein after LP implantation in 36 patients. They reported acute hemostasis in each patient. Only 1 patient had minor access site bleeding, requiring additional manual pressure and extension of flat time with 2 hours. These findings are in accordance with our results.

In contrast to closures of the femoral vein, our patients were ambulated immediately, since we used the jugular approach. Therefore, 26% of patients could be discharged the same day of implantation, which is not reported as the standard procedure when using the femoral approach [16].

Another study describing closure of the femoral vein, was the study of Regoli *et al*. [2]. In most cases (84%), 2 PP were adequately deployed after femoral vein cannulation for LP implantation in 83 patients. Additional PP were required to achieve hemostasis in 16% of cases. The authors attributed device failures to significant tortuosity of the femoral vein anatomy and also to obesity.

This is in contrast to our results, we did not experience device failures. The IJV access is less affected by obesity and tortuosity characteristics, which could explain the difference in device failures.

Regoli *et al*. [2] describe acute hemostasis of the femoral vein in 98% of patients. Major groin site complications were reported in 2 patients and vascular complications during follow up were reported in another 2 patients. These observations are in line with other studies reporting on femoral vein cannulation for large bore access for Mitraclip implantations [17, 18].

Although we did not experience jugular access site complications. This difference in complication rate could be explained by the rather superficial position of the IJV in comparison to the femoral vein, which is overlapped by the femoral artery in 92% of patients, as was shown by Randall *et al*. [19]. Surprisingly in the study by Regoli *et al*. [2] patients were instructed to lay flat for 4–6 hours, with a light pressure dressing and patients were mobilized 1–2 hours after the dressing was removed. This is in contrast to other studies using the double PP after LP implantation via the femoral approach requiring only 2–4 hours of deambulation [1]. In our study, all patients were ambulated immediately and discouraged to lay flat.

Moreover, a study by Mitacchione *et al*. [20] investigated complications in patients with LP implantation after transvenous lead extractions compared to a cohort of patients with new LP implantations in a total of 1179 patients. They reported 2.9% vascular access complications, when using the femoral approach, including femoral artery injury (1.1%) and groin hematoma (1.8%) [2, 20]. The vascular complication rate was higher compared to our results. They did not use a vascular closure device but instead a figure of 8 suture which could explain their higher vascular complication rate.

### 4.3 Internal Jugular Vein Patency

Some implanting cardiologists might have concerns about the IJV patency and diameter after large bore access. A paper by Solanki *et al*. [21] provides an excellent overview on IJV diameter measurements at different puncture locations and in various positions of the patient. They measure a diameter of 15.63 ± 3.66 mm approximately of the IJV in the supine position, which is significantly larger than the 27 Fr. (9 mm) diameter of the Micra TPS delivery sheath [21].

Regarding IJV patency, one of our patients underwent two Micra TPS implantations through the IJV with a 4-year interval between procedures, without any vascular complications. The first LP reported a short battery life due to elevated pacing thresholds and therefore a second LP was implanted 4 years later. Furthermore, we describe one patient who received an ultrasound of the IJV after implantation due to a minor hematoma to exclude vascular complications. The ultrasound did not show any vascular complications and confirmed patency of both the IJV as the carotid artery.

### 4.4 Limitations

This is a cohort study and we did not compare our results to a control group. Closure of the IJV after LP implantation with a double PP has become standard care in our hospital. Since our experience towards the PP has been favorable, we only have 2 patients in whom the IJV was closed without the use of a closure device. A major limitation of our study is that all patients were of Caucasian ethnicity, while no patients were of African descent in whom there may be a different type of scar formation.

## 5. Conclusions

Our data suggest that although the Perclose ProGlide is designed as a vascular closure device for the femoral artery and vein, it can also be safely applied for the internal jugular vein, after large bore cannulation to achieve acute hemostasis, facilitate immediate ambulation and improve patient comfort. In addition, no vascular complications were found and scar formation in the neck was minimal.

## Availability of Data and Materials

The datasets used and/or analyzed during the current study are available from the corresponding author on reasonable request.
